# Case of Tetanus, Cured by Copious Abstraction of Blood, Calomel, and Opium

**Published:** 1819-08

**Authors:** Jos. Painchaud


FOR THE LONDON MEDICAL AND PHYSICAL JOURNAL.

### *Case of Tetanus, cured by copious Abstraction of Blood, Calomel, and Opium.* By Jos. Painchaud, M.D.

On the 15th December last, Mary St. Gelais, a servant of Mr. Saul, fell on the ice, and lacerated the integuments of her right knee; but the wound not appearing dangerous, she continued her usual occupations. Eighteen days after, although the wound appeared nearly healed, she began to complain of a stiffness in the back of her neck, and of a certain difficulty in moving her jaw, accompanied with an acute pain in her knee, which the curing of the wound had not been able to dispel. The pain having increased in an alarming manner during the day, the patient was carried in the evening to Dr. Blanchet, who prescribed something for the night. Dr. A. Iffland being called in, at Mr. Saul's desire, declared that the tetanus was then complete. During three days, he employed every thing the art prescribes in such cases; but, perceiving all his efforts were useless, he requested his friend, Dr. P. de Salles Laterriere, to form a consultation. They were both of opinion that there was nothing else to be done but amputation; to which, nevertheless, the patient and her relations positively refused their consent. They then contented themselves with enlarging the wound, and dressing it with the common stimulants; leaving thus the patient with such full conviction of her approaching death, that they thought it their duty to give her warning of her extreme danger, and her relations of the certainty of her death. The disconsolate husband came to me soon after, in tears, and requested me to go and see his wife, telling me she was at the last extremity; without, however, mentioning one word of what had passed before. But, on the road, I met Dr. P. de Salles Laterriere, who informed me nearly of what I have just related, adding, that the case was quite desperate: yet the success I had already met with from copious bleedings in similar cases, prevailed on me again to try the same measures.

I found the poor woman in so violent a paroxysm, that her whole body was bent like a bow, and supported only on the back of the head and on the heels. The jaws were so closed, that it was impossible to introduce the blade of a knife. I confess that I also thought her on the very point of expiring: yet her pulse, although small and rapid, and much resembling such a one as commonly accompanies inflammation of the brain, holding out tolerably well, I immediately came to the resolution of bleeding her until she fainted. I was obliged to take from her thirty-six ounces of blood. The fainting-fit lasted a long time; but the contraction of the jaws and the general spasm yielded visibly to that powerful depletion. I then took advantage of the slackness of the jaw to make her swallow four ounces of castor-oil; and I prescribed the same quantity in a clyster. After two hours she had two copious stools: she notwithstanding relapsed, and as violently as before. I repeated the bleeding, which was followed by a fainting, after the fresh loss of eighteen ounces of blood. During three days, she took each day an ounce and a half of laudanum: the fourth day, her mouth again closed, and the same convulsions began. Another bleeding *ad deliquium*, (thirty ounces,) and the patient found herself relieved as if it were by enchantment. Her great repugnance to the tincture of opium made me substitute in its place the extract of pure opium combined with calomel: the doses will appear more than extraordinary, and the success alone can justify them.

I gave her sixty grains of opium and sixty of calomel each day, during three successive days; and, during six other successive days, sixty grains of opium alone each day. The calomel did not cause any salivation: it acted powerfully on the bowels, from which it expelled several worms. The woman is at present perfectly well, and goes about to her business.

*Quebec; 1st February,* 1819.

---

Observations *on the preceding Case, by* Dr. de Salles Laterriere; *addressed to* Dr. Painchaud.

As I understand you have perfectly succeeded in curing Marie St. Gelais, in a case of opisthotonos, and that you are inclined to publish this extraordinary cure, I am happy to have it in my power to inform you of what took place previous to your being called in. My friend, Dr. Iffland, having the patient under his care, and the disease suddenly taking a decided turn, he thought proper to consult me. He informed me that, the patient's pulse being quick and hard, he bled her, but he observed that it augmented the spasms; that he had given her a large quantity of opium, and caused her jaw to be rubbed with opiates. The morning that I saw the patient, her head was drawn entirely back, her jaw closely locked, and the spasms very frequent. We opened the wound as far as the capsular ligament of the knee, and introduced some spirits of turpentine into it, in order to excite inflammation, and, if possible, to produce suppuration; at the same time, we recommended the application of cold iced water to the head, and the patient felt some relief from it. However, notwithstanding the opium, calomel, cold water, and all that is prescribed in such cases, the disorder increased; and, after informing her and her husband of the dangerous situation in which she was, we apprised them that one thing only remained for us to try, and that was, amputation above the knee. The patient not agreeing to that, I was informed that you were sent for; and am happy to learn, that, by bleeding *ad deliquium*, you have succeeded in curing her. I am, sir, your's, &c. &c.

P. DE SALLES LATERRIERE.

*Quebec; 30th January,* 1819.

